# Comparative transcriptome analysis reveals the adaptive mechanisms of halophyte *Suaeda dendroides* encountering high saline environment

**DOI:** 10.3389/fpls.2024.1283912

**Published:** 2024-02-14

**Authors:** Panpan Ma, Jilian Li, Guoqing Sun, Jianbo Zhu

**Affiliations:** ^1^ College of Life Sciences, Shihezi University, Shihezi, China; ^2^ Xinjiang Production & Construction Group Key Laboratory of Crop Germplasm Enhancement and Gene Resources Utilization, Biotechnology Research Institute, Xinjiang Academy of Agricultural and Reclamation Sciences, Shihezi, China; ^3^ Key Laboratory of Cotton Biology and Genetic Breeding in Northwest Inland Region of the Ministry of Agriculture (Xinjiang), Institute of Cotton Research, Xinjiang Academy of Agricultural and Reclamation Sciences, Shihezi, China; ^4^ Biotechnology Research Institute, Chinese Academy of Agricultural Sciences, Beijing, China; ^5^ Western Research Institute, Chinese Academy of Agricultural Sciences, Changji, China

**Keywords:** halophyte, *Suaeda dendroides*, salt stress, transcriptome, adaptive mechanism

## Abstract

*Suaeda dendroides*, a succulent euhalophyte of the Chenopodiaceae family, intermittently spread around northern Xinjiang, China, has the ability to grow and develop in saline and alkali environments. The objective of this study was therefore to investigate the underlying molecular mechanisms of S. dendroides response to high salt conditions. 27 sequencing libraries prepared from low salt (200 mM NaCl) and high salt (800 mM NaCl) treated plants at 5 different stages were sequenced using Illumina Hiseq 2000. A total of 133,107 unigenes were obtained, of which 4,758 were DEGs. The number of DEGs in the high salt group (3,189) was more than the low salt treatment group (733) compared with the control. GO and KEGG analysis of the DEGs at different time points of the high salt treatment group showed that the genes related to cell wall biosynthesis and modification, plant hormone signal transduction, ion homeostasis, organic osmolyte accumulation, and reactive oxygen species (ROS) detoxification were significantly expressed, which indicated that these could be the main mechanisms of *S. dendroides* acclimate to high salt stress. The study provides a new perspective for understanding the molecular mechanisms of halophytes adapting to high salinity. It also provides a basis for future investigations of key salt-responsive genes in *S. dendroides*.

## 1 Introduction

Soil salinization has emerged as one of the principal abiotic stresses that threaten plant growth and development and restrict the sustainable development of modern agriculture (Lesk et al., 2016; Kopecka et al., 2023). The adverse effects of excessive soil salinity on plants mainly include osmotic and ion stress. Osmotic stress reduces water absorption of plants, resulting in water leakage. A high concentration of salt ions affects the plasma membrane and changes its permeability, which leads to excessive salt ion absorption by plants and excludes the absorption of other nutrient elements. Imbalanced ion absorption causes nutritional imbalance, inhibits growth, and produces single-salt toxicity, causing metabolic disorders (Yang and Guo, 2018; Zhao et al., 2020; Zhang et al., 2022). Understanding the physiological, biochemical, and molecular mechanisms of plant adaptation to salt stress and exploring the genes related to salt tolerance in nature is beneficial for germplasm innovation and cultivating plant varieties with improved salt tolerance. *Arabidopsis* is widely used in gene functional research and is a model plant for studying molecular mechanisms under various biotic and abiotic stresses. Much effort has been made to breed salt-tolerance crops for agricultural demand, however, the salt tolerance of these crops is limited in their systems. In contrast, halophytes, naturally grown under high salinity conditions, have evolved a series of strategies to adapt to extreme saline environments (Flowers et al., 2015; Calone et al., 2021; Rahman et al., 2021). Learning the mechanisms of salt resistance in halophytic plants and mining key salt-responsive genes are of great significance for cultivating crops with improved salt tolerance.

Halophytes are a kind of plant species that grow and complete their life cycle in saline environments where the salt concentration is greater than 200 mM NaCl, which accounts for about 1% of the world flora (Flowers and Colmer, 2008). Euhalophytes, accumulate excessive Na^+^ in their succulent leaves and stems and compartmentalize into vacuoles, enabling them to cope with high salt stress (Flowers et al., 2015). The genus *Suaeda* is a typical euhalophyte, an annual or perennial herb or sub-herb of Chenopodiaceae. It is usually distributed in coastal areas, intertidal zones, deserts, inland salt lakes, and various saline and alkali environments around the world (Wang and Song, 2019; Wang et al., 2022). The *suaeda* plants grow well with 200 mM NaCl and complete their life cycle in environments where salt and drought co-exist (Song et al., 2009; Guo et al., 2017; Guo et al., 2020). Moreover, the potential of several high salt responsive genes like transporters (*NHX1* and *HKT1*), ion channel (*AKT1*, *TPCA1*, *SLAC1*, aquaporins), antioxidant (*APX*, *CAT1*, *GST*) and osmotic (*CMO*, *BADH* and *P5CS*) have been identified and cloned (Mishra and Tanna, 2017), and some of these genes were explored for developing stress tolerance in the glycophyte (Lv et al., 2008; Wu et al., 2008; Shao et al., 2014; Himabindu et al., 2016; He et al., 2017; Hao et al., 2020). In addition, studies have increasingly found that halophytes can be economical and environmentally friendly candidate species for phytoremediation; excessive salt and heavy metals can be removed from salt and contaminated soils through various strategies to support plant growth (Liang and Shi, 2021; Wang L. et al., 2021). The above research shows that halophytes have important ecological and economic values. A comprehensive study on salt tolerance mechanism of halophytes is crucial for excavating key genes and cultivating stress-resistant varieties, as well as phytoremediation, which is conducive to promoting the sustainable development of agricultural economy in saline-alkali areas.

*Suaeda* plants can adapt to high concentration saline soil and act as ideal model plants for investigating complicated physiological and molecular mechanisms of salt tolerance. To explore the molecular mechanism of *S*. *dendroides* adapting to salt stress conditions, two groups of *de novo* assembled data were generated from mRNA sequencing of shoot samples from low salt and high salt treated seedlings. Bioinformatics analyses were adopted to compare changes at mRNA levels. These data would contribute to elucidating the molecular mechanisms of *S. dendroides* acclimate to high salt stress. Furthermore, the transcript information of *S. dendroides* under high salt treatment was obtained comprehensively, which provided a basis for studying gene expression levels, discovering new transcripts, and exploring the functions of key salt-tolerant genes.

## 2 Materials and methods

### 2.1 Plant materials and NaCl treatment

The seeds of *S. dendroides* were collected from saline soil in Fuhai, Xinjiang Uygur Autonomous Region, China. *S. dendroides* seeds were sown in plastic pots (12×12 cm), filled with mixed matrix (sand:vermiculite:nutritive soil = 10:1:2, w/w/w) and irrigated with tap water, placed in a plant culture room, maintained a day/night thermoperiod of 25/20°C, a photoperiod of 16 h light/8 h dark, relative humidity of (50 ± 10)%. 10 seedlings with uniform growth were maintained per pot after germination and irrigated with tap water once a week. After one month of incubation, the seedlings were irrigated with 0, 200, 400, 600, 800, and 1,000 mM NaCl solutions. 150 mL of the NaCl solution was applied in salt treatments, while the control pots were irrigated with the same volume of tap water. Throughout the experimental process, water was replenished daily through weighing methods to ensure that the water content of each pot was maintained at 50%.

### 2.2 Determination of morphological and physiological characters

According to the growth and phenotype of the *S. dendroides* seedlings under different concentrations of salt stress, the physiological and biochemical parameters were determined after treatment for 10 days. Each variable was determined in three biological replicates.

The shoot samples were taken for biomass and ion content analyses. The fresh weight (FW) of aerial tissues was measured immediately after harvest. The dry weight (DW) was determined by putting the FW samples in an oven at 105 °C for 10 min, then drying for 48 h at 85°C until the mass was constant. Relative water content (RWC) was calculated using the formula: RWC = (FW-DW)/FW×100%. In order to know the salt absorption of *S. dendroides* plants, Na content in the seedlings under salt treatment was detected. Briefly, 100 mg dried powder from the shoot of each treatment sample was weighed and digested with 5 mL HNO_3_ overnight and incubated at 80°C for 1~2 h, 120°C for 1~2 h, then 160°C for 4 h, cooling to room temperature, after filtering the extracts, Na and K content was determined by inductively coupled plasma atomic emission spectroscopy (ICP-AES/OES/MS).

Fresh leaf samples were taken for physiological and biochemical parameters analyses, such as chlorophyll content, ribulose-1,5-bisphosphate carboxylase/oxygenase (Rubisco) activity, malondialdehyde (MDA) content, proline content, soluble sugar content. All the above variables were assayed as the instructions of the reagent kit (Solarbio, China), and the absorbance was measured using a UV2300 ultraviolet visible spectrophotometer (Tianmei, China).

### 2.3 RNA sequencing and bioinformatics analysi*s*

**Figure 1:**
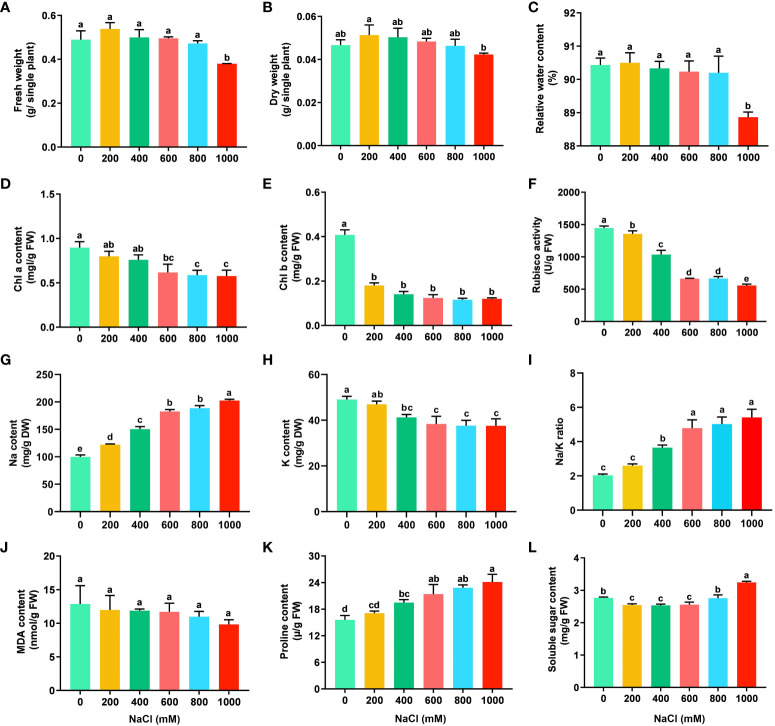
Physiological variables of *S. dendroides* under salt stress. **(A)** Fresh weight, **(B)** Dry weight, **(C)** Relative water content, **(D)** Chl a content. **(E)** Chl b content, **(F)** Rubisco activity, **(G)** Na content, **(H)** K content, **(I)** Na/K ratio, **(J)** MDA content, **(K)** Proline content, **(L)** Soluble sugar content. X-axis means the concentration of NaCl (mM). Statistical analysis was conducted using SPSS 20.0 software, the significant difference between different treatments was obtained through Tukey’s test of one-way ANOVA analysis. Values are presented as the mean ± the standard error (SE) with three biological replicates. The different letters above the bars indicate the least significant differences between treatments at p < 0.05.

Based on our preliminary physical investigations, 200 mM NaCl is the optimal salt concentration, and 800 mM NaCl is the highest salt concentration for the growth of *S. dendroides* seedlings (**Figure 1**). Consequently, the shoot samples were harvested at 0 (before the treatment, marked as C), 1, 5, 10, and 15 days after 200 (marked as L1, L5, L10, and L15) and 800 mM NaCl treatments (marked as H1, H5, H10, and H15) for RNA- sequencing and QPCR. Total RNA extraction from 27 shoot tissues of *S. dendroides* was according to the manufacturer’s procedure of the EASYspin Plus Plant RNA Kit (Aidlab, China). Qubit^®^ RNA Assay Kit in Qubit^®^ 2.0 Fluorometer (Life Technologies, USA) was used to detect RNA concentration, and RNA Nano 6000 Assay Kit of Agilent Bioanalyzer 2100 system (Life Technologies, USA) was used to evaluate RNA integrity. NEBNext^®^ Ultra™ RNA Library Prep Kit of Illumina^®^ (NEB, USA) was used to generate sequencing libraries following the manufacturer’s recommendations, and library quality was evaluated by the Agilent Bioanalyzer 2100 system. The libraries were sequenced on an Illumina Hiseq platform, and paired-end reads were generated. After filtering adaptor sequences, duplicated sequences, low-quality bases, and undetermined bases, the high-quality clean reads were assembled using Trinity software (Grabherr et al., 2011). Function annotation of the assembled unigenes was carried out through NCBI non-redundant protein sequences (Nr), NCBI non-redundant nucleotide sequences (Nt), Protein family (Pfam), Clusters of Orthologous Groups of proteins (KOG/COG), A manually annotated and reviewed protein sequence database (Swiss-Prot), KEGG Ortholog database (KO) and Gene Ontology (GO) databases.

### 2.4 DEG selection and functional enrichment analysis

The transcription level of the assembled unigenes was evaluated according to FPKM to identify DEGs between L1 vs C, L5 vs C, L10 vs C, L15 vc C, H1 vs C, H5 vs C, H10 vs C, H15 vs C, H1vs L1, H5 vs L5, H10 vs L10, and H15 vs L15 compares. Differential expression analysis of two conditions/groups was carried out by DESeq2 software (Love et al., 2014). The negative binomial Wald test was used as a statistical test, and then the Benjamini-Hochberg correction was used to obtain the false discovery rate (FDR) (Benjamini and Hochberg, 1995; Love et al., 2014). FDR < 0.05 and |log2FC| > 1 were set as the thresholds for screening of significantly differential expressed genes. VENNY 2.1 (https://bioinfogp.cnb.csic.es/tools/venny/), a free online platform, was used to draw Venn diagrams. TBtools v1.120 was used to construct the heatmaps for gene expression. GO and KEGG enrichment analysis of DEGs were performed using GOseq R packages based on Wallenius non-central hyper-geometric distribution (Young et al., 2010) and KOBAS software (Mao et al., 2005). The sequences of the target DEGs were aligned to the protein sequence of the reference species contained in the STRING database (http://string-db.org/) by using blasx, and an interaction network was constructed by using the protein-protein interaction (PPI) relationship of the reference species. The PPI network of the DEGs was visualized and edited by Cytoscape software v3.9.1 (Shannon et al., 2003).

### 2.5 Gene expression validation using quantitative real-time PCR

10 putative DEGs involved in salt conditions were randomly selected for validation of differential expression using QPCR. The primers for the selected genes were designed according to the sequences assembled by *S. dendroides* RNA sequencing and are listed in **Supplementary Table S1**. 18S gene of *Anabasis aphylla* was used as the reference for quantitative expression analysis. 1 ug RNA of 27 samples was reverse transcribed into cDNA with oligo (dT) primers, and the cDNA libraries were used for QPCR. A total of 20µL fluorescent quantitative reaction system contains 10 µL of 2 × PerfectStart^®^ Green qPCR SuperMix (TransGen, China), 2 µL of cDNA template, 0.8 µL of 10 mM primers and 0.4 μL of 50 × ROX Reference Dye. QPCR was performed on an ABI 7500 platform, and the settings were as follows: step 1 95°C, 30 s; step 2 95°C, 5 s, 60°C, 34 s, 40 cycles, followed by the melting curve analysis. Each DEG was analyzed in three biological samples, and each biological sample was repeated three times. 2^-^ *^ΔΔ^* ^CT^ method was used to calculate the relative expression levels of the target genes (Livak and Schmittgen, 2001).

### 2.6 Statistical analysis

Statistical analysis was conducted using SPSS 20.0 software, the significant differences were analyzed by one-way ANOVA and Tukey’s test (p < 0.05). The images were visualized using GraphPad Prism software (v. 8.3.0.538), and the data was shown as the mean ± standard deviation (SD) value of at least three biological replicates.

## 3 Results

### 3.1 Phenotypic and physiological indicators of *S. dendroides* under salt stress

The physiological and biochemical features of *S. dendroides* seedlings under different salt concentrations were performed to estimate the optimal and highest salt concentration for their growth. The seedlings of *S. dendroides* did not show symptoms of salinity injury after being treated with 200, 400, and 600 mM NaCl treatment compared with the control. The seedlings showed wilting symptoms under 800 and 1,000 mM NaCl treatment and began to recover after 5 days and recovered after 10 days, then gradually returned to normal growth. Under 1000mM NaCl stress, although some seedlings died, most seedlings survived, and the wilting symptoms slowly recovered (**Supplementary Figure S1**). The fresh weight, dry weight, and relative water content of seedlings showed the highest under 200 mM NaCl treatment, but the difference was not significant compared with the control. However, the fresh weight, dry weight, and relative water content of seedlings treated with 1000 mM NaCl were significantly lower than those of other salt concentrations (**Figures 1A-C**). The results indicate that a low concentration of NaCl could promote the growth of seedlings. The content of Chl a and Chl b, and Rubisco activity decreased obviously with the increase of NaCl concentration (**Figures 1D-F**), respectively. Na and K content in the shoots of *S. dendroides* were determined, and the results showed that Na content was significantly increased with the increase of NaCl concentration. When the NaCl concentration reached 400 mM, the accumulation of Na was 1.5 times that of the control (**Figure 1G**). The content of K decreased obviously with the increase of salt concentration, which was opposite to Na content (**Figure 1H**). The trend of Na/K ratio was consistent with that of Na accumulation. When the salt concentration increased to 600 mM, the change of Na/K ratio was moderate and kept a relatively stable level (**Figure 1I**). The results indicated that *S. dendroides* could maintain Na/K balance under high salt stress to avoid ion toxicity.

The physiological parameters, such as MDA, proline, and soluble sugar, are usually used to evaluate salt tolerance of plants. In *S. dendroides*, MDA content decreased gradually with the increase of NaCl concentration, but the difference was not significant (**Figure 1J**), indicating that salt in this concentration range did not cause the cell membrane lipid peroxidation of *S. dendroides*. The content of free proline in the seedlings of *S. dendroides* was significantly increased with the increase of NaCl concentrations (**Figure 1K**). The soluble sugar content decreased significantly under 200, 400 and 600 mM NaCl treatment, but it was significantly higher than the control when the salt concentration increased to 1000 mM (**Figure 1L**).

### 3.2 RNA-seq, *de novo* assembly and unigene functional annotation

RNA-seq analyses on the *S. dendroides* with water (C), 200 mM NaCl (L), and 800 mM NaCl (H) treatments at different time points (1, 5, 10, and 15 days) were performed. In total, 27 cDNA libraries were generated and sequenced. A total of 211.13 Gb clean data were obtained, and each sample library yielded a mean size of 7.8 Gb clean data, a Q30 percentage higher than 93.65%, and a GC percentage of 42% (**Supplementary Table S2**). Pearson’s coefficient of pairwise biological replicates was greater than 0.85, indicating that the consistency among the replicates was high (**Supplementary Figure S2**). The data shown above demonstrate that the transcriptome sequencing is of high quality. Since *S. dendroides* has no reference genome, Trinity software was used to do the *de novo* assembly and get the reference sequence for subsequent analysis. 133,107 unigenes were obtained from 27 libraries. The size distribution was shown in **Supplementary Figure S3**, the sequence of 38,197 unigenes was longer than 1,000 bp, and 12,286 unigenes was longer than 2000 bp, indicating that some candidate genes have obtained nearly full-length sequence. The mean sequence length of all unigenes was 918 bp, and the N50 length was 1,240 bp (**Supplementary Table S3**).

133,107 unigene sequences of *S. dendroides* were aligned based on similarities to the NR, NT, Pfam, Swiss-Prot, KEGG, GO, and KOG databases. The annotation results were shown in **Supplementary Table S4**, all 133,107 (100%) unigene sequences were annotated at least in one database, and 6,443 (4.84%) aligned to all seven databases. A total of 58,336 (43.82%), 42,308 (31.78%), and 42,637 (32.03%) unigenes had significant BLAST matches in NR, NT, and Pfam databases, respectively. Moreover, 42,637 (32.03%) unigenes were annotated in at least one term of three GO categories (**Supplementary Figure S4**), 19,118 (14.36%) unigenes were annotated in the KEGG database and classified into 130 KEGG pathways (**Supplementary Figure S5**). In addition, 11,489 (8.63%) unigenes were annotated in the KOG database and divided into 25 functional categories (**Supplementary Figure S6**). By comparing the annotation results with the NR database, the distribution of species on the unigene comparison was statistically analyzed. The result showed that *S. dendroides* transcriptome sequences were well matched with the species of *Chenopodium quinoa* (29.8%), *Beta vulgaris* (27.8%), and *Spinacia oleracea* (19.0%) belonging to Amaranthaceae (**Supplementary Figure S7**).

### 3.3 DEGs in *S. dendroides* under salt treatment

**Figure 2:**
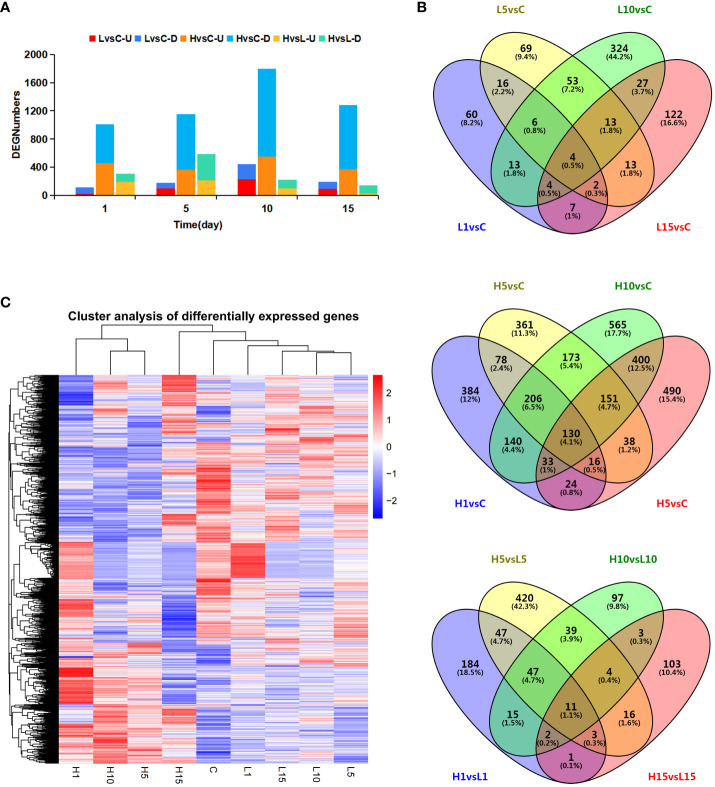
The numbers of all DEGs at different salt-stressed time points of two NaCl concentrations. **(A)** Numbers of DEGs in pairwise comparisons at different salt-stressed time points of two NaCl concentrations, **(B)** Venn diagram showed the number of DEGs in different treatment, **(C)** Heatmap diagrams showed the cluster analysis of DEGs among the treatments. Number of DEGs (FDR< 0.05 and |log 2 Fold Change| > 1) under 200mM (L) and 800mM (H) NaCl stress for 1, 5,10 and 15 day as compared to control samples.

DEGs under salt-stressed conditions were defined according to threshold FDR < 0.05 and |log_2_ fold change| > 1 among different salt-treated samples. A total of 733, 3189, and 992 DEGs were detected between L vs C, H vs C, and H vs L pairwise comparisons, respectively. DEGs were analyzed at each time interval after NaCl treatment (**Figure 2A**). With the extension of salt treatment time, the number of DEGs increased to 444 and 1,796 after 10 days, which was the peak of salt-responsive genes under both NaCl concentrations, indicating that this period was the key time for *S. dendroides* to adapt to the salt environment. Down-regulated DEG numbers were more than up-regulated genes among all the treatments. 130 of the 3,189 DEGs were detected among different times of 800 mM NaCl treatment. In addition, only 4 DEGs were detected among 733 DEGs at different times of 200 mM NaCl treatment (**Figure 2B**). In addition, the expression profiles of DEGs under each treatment were visualized in the heat map (**Figure 2C**). The expression patterns of most DEGs of samples H1, H5, and H10 were similar and clustered in one class, while the samples H15, C, L1, L5, L10, and L15 were clustered in another class. The results showed that the expression pattern of DEGs under 200 mM NaCl treatment was similar to the control, while the DEGs under 800 mM NaCl treatment were contrary to the control. These results indicated that the euhalophyte *S. dendroides* gradually adapted to the high salt stress environment, in which the 10 days was a critical time for *S. dendroides* to cope with the high salt stress environment. Consequently, the following analysis focuses on the DEGs of 800 mM NaCl treatment to get a comprehensive understanding of the adaptive mechanisms of *S. dendroides* encountering high salt stress.

**Figure 3:**
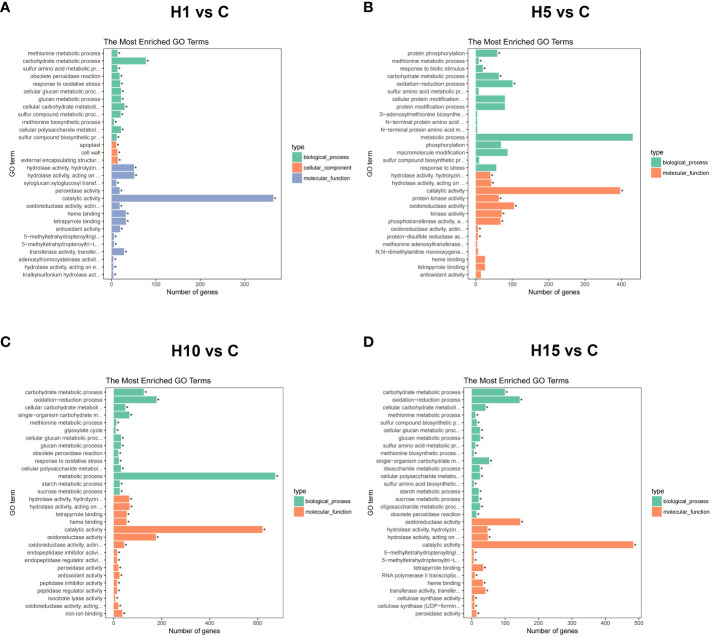
GO enrichment analysis of DEGs in **(A)** H1 vs C, **(B)** H5 vs C, **(C)** H10 vs C, **(D)** H15 vs C comparisons of high salt stress. Top 30 GO enrichment terms of differently expressed genes significant p-values (p < 0.05). GO terms are mainly divided into three classes: cellular component, molecular function, and biological process. X-axis means the number of DEGs. Y-axis represents GO terms.

**Figure 4:**
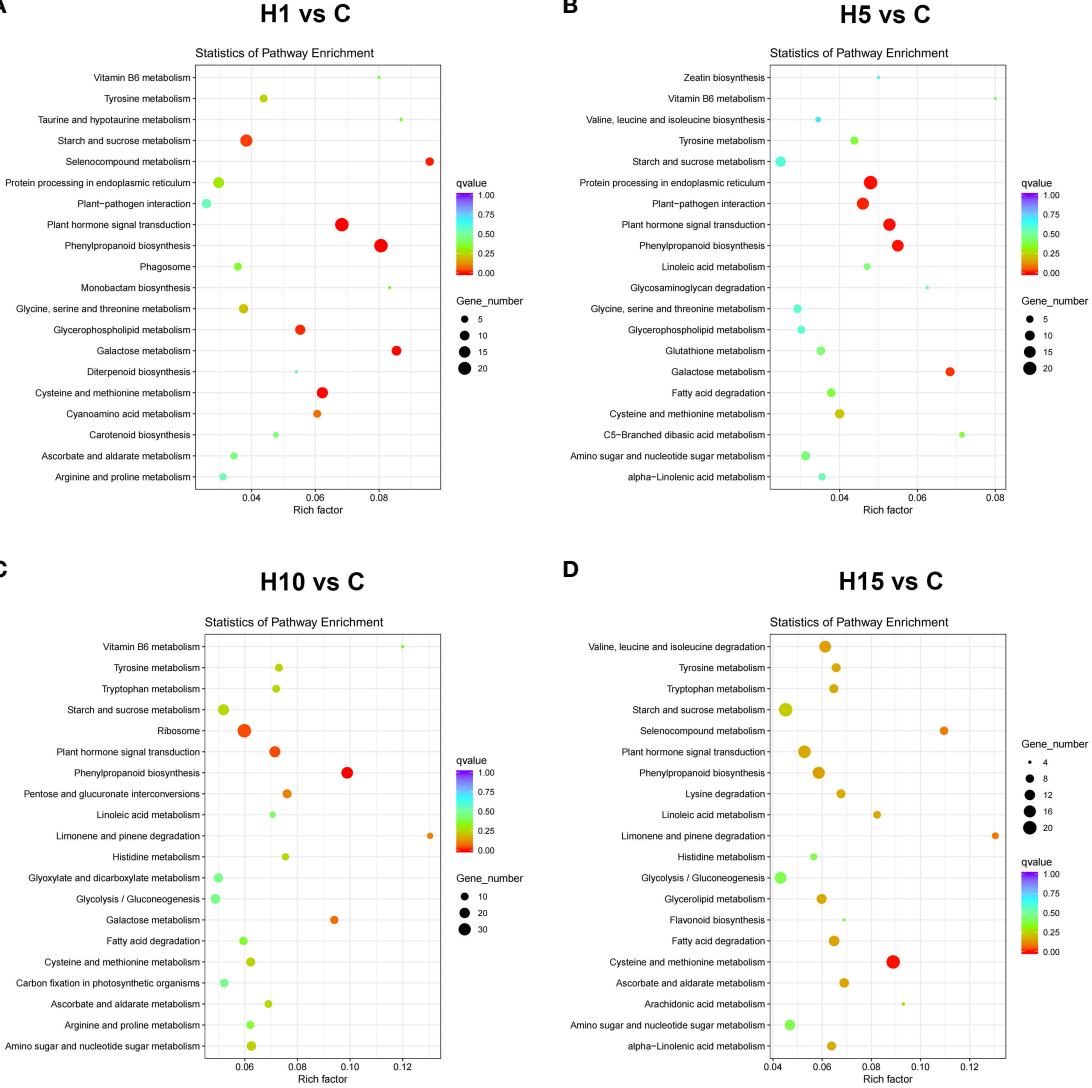
KEGG pathway enrichment analysis of DEGs in **(A)** H1 vs C, **(B)** H5 vs C, **(C)** H10 vs C, **(D)** H15 vs C comparisons of high salt stress. Top 20 GO enrichment terms of differently expressed genes significant p-values (p < 0.05). X-axis represents Rich factor. Y-axis represents the KEGG pathway.

GO and KEGG enrichment analyses were carried out on DEGs at different time points with high salt treatment. GO enrichment analysis showed that the DEGs of H1 vs C, H5 vs C, H10 vs C, and H15 vs C comparisons were significantly enriched in 45, 14, 41, and 39 processes (FDR < 0.05), respectively (**Supplementary Table S5**). The top 30 Go items of different salt time points were shown in **Figures 3A-D**, the catalytic activity item was notably enriched at each salt time point, and the DEG numbers were the highest. Besides, carbohydrate metabolic, cell metabolic, methionine metabolic, protein phosphorylation and protein kinase were the most enriched items at each salt treatment. KEGG enrichment results showed that the DEGs of H1 vs C, H5 vs C, H10 vs C, and H15 vs C comparisons were significantly enriched (p-value < 0.05) in 11, 9, 13 and 16 pathways, respectively (**Supplementary Table S6**). Among these, phenylpropane metabolism, plant hormone signal transduction, cysteine and methionine metabolism, glycerophospholipid metabolism, starch and sucrose metabolism, and ribosome pathway at different salt treatment time points were significantly enriched (**Figures 4A-D**; **Supplementary Table S6**). In addition, cell wall-related metabolic pathways were also found in this study, such as the galactose metabolic pathway closely related to pectin, the pentose glucuronic acid conversion, and the flavonoid biosynthesis metabolism closely related to lignin, the phenylalanine metabolic pathway, and keratin and wax biosynthesis. Moreover, the key protein-protein interaction (PPI) network of high NaCl treatment for 10 days was predicted. The result showed that DEGs related to cell wall synthesis and modification, ABA signaling, ethylene biosynthesis, and organic osmolyte accumulation under high salt treatment, which is consistent with GO and KEGG enrichment results. The above results indicate that cell wall synthesis and modification, ABA signaling, ethylene biosynthesis and signal, organic osmolyte accumulation, and ROS homeostasis might be the main adaptation mechanisms of *S. dendroides* suffering from extreme salt stress.

### 3.4 DEGs related to cell wall synthesis and modification under high salt treatment

**Figure 5:**
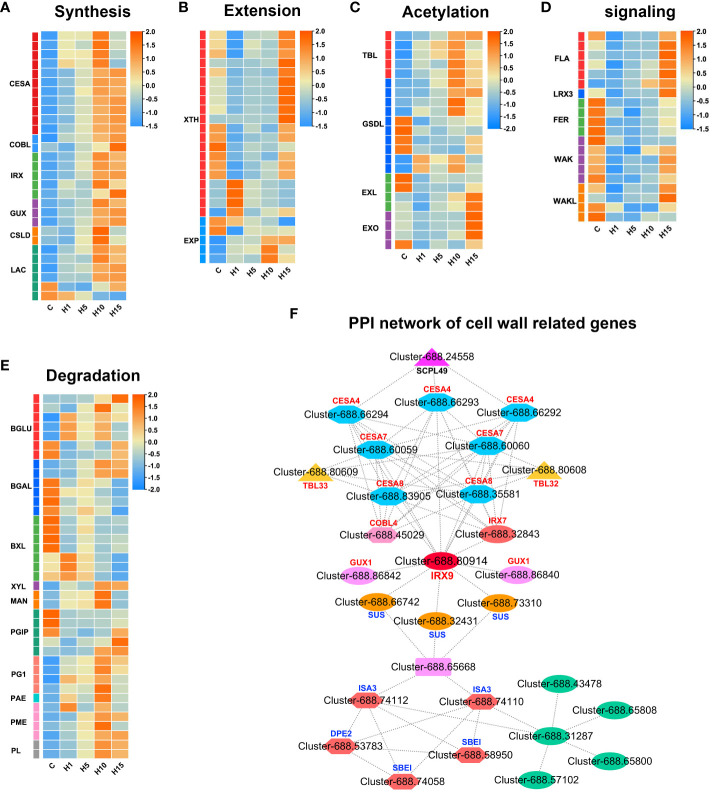
Expression profiles of DEGs related to cell wall **(A)** synthesis, **(B)** extension, **(C)** acetylation, **(D)** proteins and signaling, **(E)** degradation, **(F)** PPI network of cell wall related DEGs in *S. dendroides* after 10 days of high salt stress. The column names C, H1, H5, H10, and H15 in each heat map are the samples treated with 800 mM NaCl after 0, 1, 5, 10, and 15 days. The expression level is represented by the mean value of FPKM (n=3), and the color scale in the upper right represents the normalized FPKM value.

**Figure 6:**
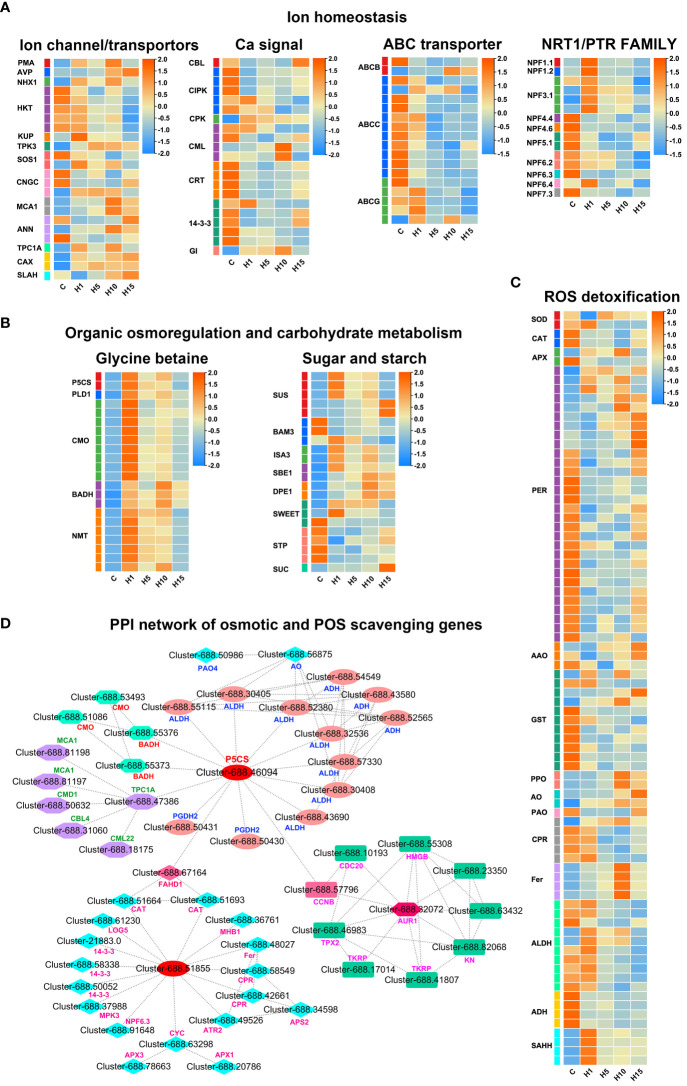
Expression profiles of DEGs related to **(A)** Ion homeostasis, **(B)** Organic osmoregulation and carbohydrate metabolism, **(C)** ROS detoxification, **(D)** PPI network of osmotic and ROS scavenging related DEGs in *S. dendroides* after 10 days of high salt stress. The column names C, H1, H5, H10, and H15 in each heat map are the samples treated with 800 mM NaCl after 0, 1, 5, 10, and 15 days. The expression level is represented by the mean value of FPKM (n=3), and the color scale in the upper right represents the normalized FPKM value.

According to GO, KEGG enrichment, and PPI results, the DEGs involved in cell wall metabolism of *S. dendroides* under high saline conditions were analyzed. 13 DEGs involved in cellulose synthesis were induced by 800 mM NaCl stress (**Figure 5A**; **Supplementary Table S7**). Among these, 4 predicted cellulose synthase A (*CESA*) genes, including 1 *CESA1* and 3 *CESA3* genes, which produced cellulose in the primary cell wall (PCW), were up-regulated and significantly increased after 10 days of treatment. Moreover, 3 predicted *CESA4*, 2 xylem-specific *CESA7*, and 2 *CESA8* genes involved in the biosynthesis of the secondary cell wall (SCW) were up-regulated and dramatically increased after 10 and 15 days of 800 mM NaCl treatment. In addition, 2 COBRA-like (*COBLs*) genes related to cell wall biosynthesis were up-regulated under 800 mM NaCl treatment and prominently increased after 15 days of the treatment. Moreover, several genes involved in hemicellulose synthesis were detected under high salt stress. 6 glycosyltransferase (GT) family genes involved in the biosynthesis of xylan backbone were identified under high salt treatment (**Figure 5A**; **Supplementary Table S7**). Such as glucuronoxylan glucuronosyltransferase (*IRX7*), beta-1,4-xylosyltransferase (*IRX9* and *IRX14*), galacturonosyltransferase-like 1 (*GATL1*), UDP-glucuronate:xylan alpha-glucuronosyltransferase 1 (*GUX1*/*PGSIP*1 and *GUX2*/*PGSIP3*) related to the secondary cell wall biosynthesis, were all significantly increased after 10 days of 800 mM NaCl stress. Meanwhile, 2 non-glycosyltransferase genes, IRREGULAR XYLEM 1 (*IRX15*) and IRREGULAR XYLEM 1 like (*IRX15L*), related to xylan backbone synthesis were up-regulated under high salt treatment. 2 cellulose synthase-like protein D5 (*CLSD5*) related to the biosynthesis of hemicellulose polysaccharides were detected to be significantly expressed under 800 mM NaCl stress. In addition, 6 laccases (*LAC*) and 30 peroxidases (*PER*) genes related to the polymerization of lignin monolignol were also found under high salt stress (**Figures 5A**, **6C**; **Supplementary Table S7**).

Interestingly, multiple DEGs involved in cell wall modification and remodeling were identified under 800 mM NaCl stress. 20 xyloglucan endotransglucosylase/hydrolase (*XTH*/*XET*) and 5 expansins/expansins-like (*EXP*/*EXL*) genes that participated in cell wall loosening were identified (**Figure 5B**; **Supplementary Table S7**). Several glycosidase encoding DEGs involved in hydrolyzing hemicellulose polysaccharides were obtained, such as 7 β-galactosidase (*BGLU*), 6 β-galactosidase (*BGAL*), and 7 β-D-xylosidases (*BXL*) genes were differentially expressed under 800 mM NaCl stress (**Figure 5E**; **Supplementary Table S7**). *XYL* involved in xyloglucan degradation was down-regulated under high salt treatment. Additionally, pectin metabolic enzymes (**Figure 5E**; **Supplementary Table S7**), including pectin acetylesterase (*PAE*), pectin methyl esterase (*PME*), polygalacturonase (*PG*), polygalacturonase inhibitor (*PGIP*), and pectate lyase (*PL*) encoding genes involved in cell wall remodeling were obtained under high salt treatment. 4 *PMEs*, 2 *PLs*, 4 *PG1s*, and 1 *PAE* were up-regulated under 800 mM NaCl treatment (**Figure 5E**; **Supplementary Table S7**). 5 trichome birefringence-like (*TBL*) genes involved in xylan acetylation were up-regulated, 6 GDSL esterase/lipase (*GSDL*) encoding genes that affecting xylan deacetylation were up-regulated under high salt stress, especially after 10 days, 6 EXORDIUM/EXORDIUM-like (*EXO/EXL*) genes involved in the regulation of secondary cell wall thickening and lignification were significantly increased after 15 days of high salt stress (**Figure 5C**; **Supplementary Table S7**). Moreover, several cell wall genes and receptor-like kinase genes were differentially expressed under high salt treatment (**Figure 5D**; **Supplementary Table S7**). 6 DEGs encoding FASCICLIN-like arabinogalactan protein (FLA) were notably increased after 15 days of high salt treatment. 1 leucine-rich repeat extensin-like protein (LRX3) decreased at the early stage of high salt treatment and increased after 15 days. 4 FERONIA (*FER*) genes of *Catharanthus roseus* RLK1-like kinase (*CrRLK1L*) family were down-regulated under high salt stress. 9 Wall-associated receptor kinase (*WAK/WAKL*) encoding genes were decreased at the early stage of high salt treatment and increased to varying degrees after 15 days of 800 mM NaCl treatment.

### 3.5 DEGs related to ion transport and Ca^2+^ signaling

Several DEGs associated with ion transport were obtained under 800 mM NaCl treatment in the study (**Figure 6A**; **Supplementary Table 7**). A sodium/hydrogen exchanger protein NHX1 involved in vacuolar Na^+^ transport was significantly up-regulated under 800 mM NaCl treatment. Similarly, a two-pore potassium channel protein TPK3 associated with vacuolar K^+^ transport, 2 vacuolar Ca^2+/^H^+^ exchanger (*CAX*) genes were significantly up-regulated. DEGs of plasma membrane ATPase (*PMA*) and vacuolar H^+^-pyrophosphatases (*AVP*) were also up-regulated. The results indicated that the up-regulated *NHX*, *PMA*, and *AVP* might be crucial for Na^+^ sequestration into the vacuolar of *S. dendroides*. 5 high-affinity K^+^ transporter (HKT1) encoding genes involved in Na^+^ transport were obtained, the expression level of 3 genes was significantly increased after 1 day, and 2 were increased significantly after 15 days of high salt treatment. Potassium transporter (KUP) was significantly up-regulated after 1 day of 800 mM NaCl treatment. 3 cyclic nucleotide-gated ion channel (*CNGC*) genes were obtained, *CNGC4* was up-regulated, and 2 *CNGC17* genes were down-regulated under high salt treatment. 2 Ca^2+^ influx channel proteins mid1-complementing activity 1 (MCA1) were up-regulated, especially after 10 and 15 days of 800 mM NaCl treatment. The increasing cytoplasmic Ca^2+^ are decoded by a series of Ca^2+^ sensors or binding proteins, such as calcineurin B-like protein (CBL), Ca^2+^- dependent protein kinases (CPK), Calmodulin (CaM), calmodulin-like proteins (CML), calcium-dependent protein kinase (CIPK) and calreticulin (CRT). 21 DEGs that participated in Ca^2+^ signaling pathway were obtained, as shown in **Figure 6A**, *CBL4, CIPKs*, *CMLs*, and *CRTs* were induced by high salt stress. Several nitrate transporter 1/peptide transporter, NRT1/PTR family (NPF) encoding genes related to NO^3−^/Cl^-^ transport were obtained (**Figure 6A**). Among these, 8 NPF genes were down-regulated, and 7 were significantly up-regulated after 1 day of high salt stress. Besides, 18 ABC transporters were differently expressed under high salt concentration treatment, in which 3 *ABCC22* genes were notably increased after 1 day, 3 genes were up-regulated at all the treatment times, while the remaining 12 genes were down-regulated at all the treatments.

### 3.6 DEGs related to organic osmolyte synthesis and ROS detoxification

The accumulation of organic adjustment substances in succulent leaves is another important reason for *S. dendroides* adapting to high salt environment. In this study, 44 DEGs positively correlated with the synthesis of organic adjustment substances proline, betaine, and soluble sugar were identified (**Figure 6B**). Among these, 2 delta-1-pyrroline-5-carboxylate synthase (*P5CS*), a key enzyme gene involved in proline synthesis, were significantly up-regulated under 800 mM NaCl treatment. 9 choline monooxygenase (*CMO*) and 3 betaine aldehyde dehydrogenase (*BADH*), key enzyme genes participated in betaine synthesis, were also significantly up-regulated. Moreover, 7 phosphoethanolamine N-methyltransferase (*NMT*) genes were notably up-regulated after 1 day of high salt treatment. Likewise, 5 sucrose synthase (*SUS*) encoding genes and 2 4-alpha-glucanotransferase (*DPE1*) involved in soluble carbohydrate accumulation were significantly up-regulated under 800 mM NaCl treatment. 1 sucrose transport protein SUC and 2 bidirectional sugar transporters (SWEET) were significantly increased under high salt stress. These results showed that the up-regulated DEGs of organic osmolyte synthesis under high salt treatment, including proline, betaine, and soluble sugar, play vital roles in osmoregulation of *S. dendroides*. Antioxidant enzymes and non-enzymatic compounds were crucial for detoxification of ROS under stress conditions. In *S. dendroides*, the genes of ascorbate peroxidase (*APX*), superoxide dismutase (*SOD*), catalase (*CAT*), *PER*, glutathione S-transferase (*GST*), and L-ascorbate oxidase (*AAO*) were significantly enriched and differentially expressed, to avoiding oxidative stress induced by high salinity (**Figure 6C**). Besides, polyphenol oxidase (*PPO*), primary amine oxidase (*AO*), and ferritin (*Fer*) encoding genes were up-regulated at all time points after 800 mM NaCl treatment. 10 aldehyde dehydrogenase (*ALDH*) encoding genes and 4 alcohol dehydrogenase-like (*ADH*)encoding genes were differently expressed (**Figure 6C**).

### 3.7 DEGs related to hormone synthesis and signal transduction in response to high salt stress

**Figure 7:**
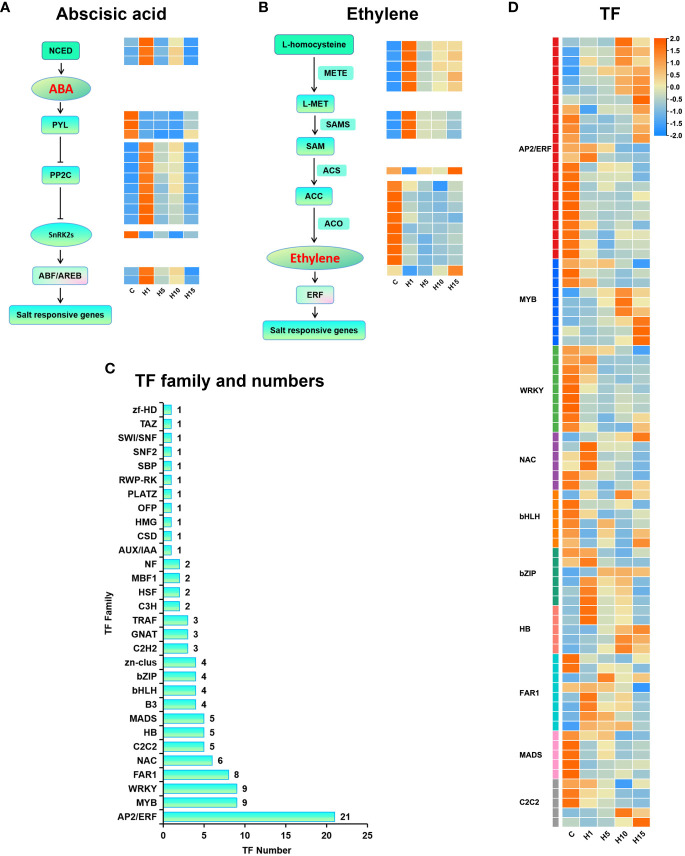
Expression profiles of the plant hormone signal transduction genes and TFs of *S. dendroides* in response to high salt treatment. **(A)** ABA synthesis and signal transduction genes, **(B)** ETH synthesis and signal transduction genes, **(C)** The numbers of different transcription factor families, **(D)** Expression profiles of TFs. The column names C, H1, H5, H10, and H15 in each heat map are the samples treated with 800 mM NaCl after 0, 1, 5, 10, and 15 days. The expression level is represented by the mean value of FPKM (n=3), and the color scale in the upper right represents the normalized FPKM value.

GO and KEGG enrichment results showed that the DEGs under high salt stress were significantly enriched in plant hormone signal transduction pathways like ABA and ethylene signal transduction. Several DEGs that participated in ABA signal transduction were differentially expressed under high salt treatment (**Figure 7A**). 3 9-cis-epoxycarotenoid dioxygenase (*NCED*) encoding genes, involved in ABA synthesis, were rapidly up-regulated after 1 day of high salt treatment. 3 genes encoding ABA receptor *PYLs* were detected, *PYR1* and *PYL4* were rapidly down-regulated under 800 mM NaCl treatment. 9 protein phosphatase 2C (*PP2C*) encoding genes were prominently up-regulated after 1 day of 800 mM NaCl treatment. 1 SNF1-related protein kinases (*SnRK2*) gene was down-regulated under 800 mM NaCl treatment. 2 ABA-responsive element binding factors (*ABF2*) were increased dramatically after 1 day and declined after 15 days of 800 mM NaCl treatment. 27 DEGs enriched in cysteine and methionine metabolic pathway were mainly associated with ethylene biosynthesis (**Figure 7B**), such as 5-methyltetrahydropteryltriglutamate homocysteine methyltransferase (*METE*), S-adenosylmethionine synthase (*SAMS*), 1-aminocyclopropane-1-carboxylic acid synthase (*ACS*), 1-aminocyclopropane-1-carboxylic oxidase (*ACO*) and other key enzymes in ethylene biosynthesis. 5 *METE* genes and 3 *SAMS* genes were up-regulated under 800 mM NaCl treatment. 9 *ACO* and *ACO* homologs genes were down-regulated.

### 3.8 Expression profiling of transcription factors associated with salt tolerance

Transcription factors (TFs) play a key role in regulating plant resistance to abiotic stress. In this study, 1,864 TF members were obtained in the assembly transcriptome sequence of *S. dendroides*, and 120 members were differently expressed under high salt treatment. In addition, 26, 36, 64, and 63 TFs were differently expressed in H1 vs C, H5 vs C, H10 vs C, and H15 vs C comparisons, respectively (**Supplementary Table S8**). The AP2/ERF family is the most abundant TF family in each comparison, followed by WRKY, MYB, FAR1, NAC, MADS, HB, bHLH, and bZIP (**Figure 7C**), suggesting that these TFs might be the main regulatory factors of *S. dendroides* plants under high salt treatment. The expression profiles of these major TFs under high salt treatment are displayed in **Figure 7D**.

### 3.9 QPCR validation of DEGs

**Figure 8:**
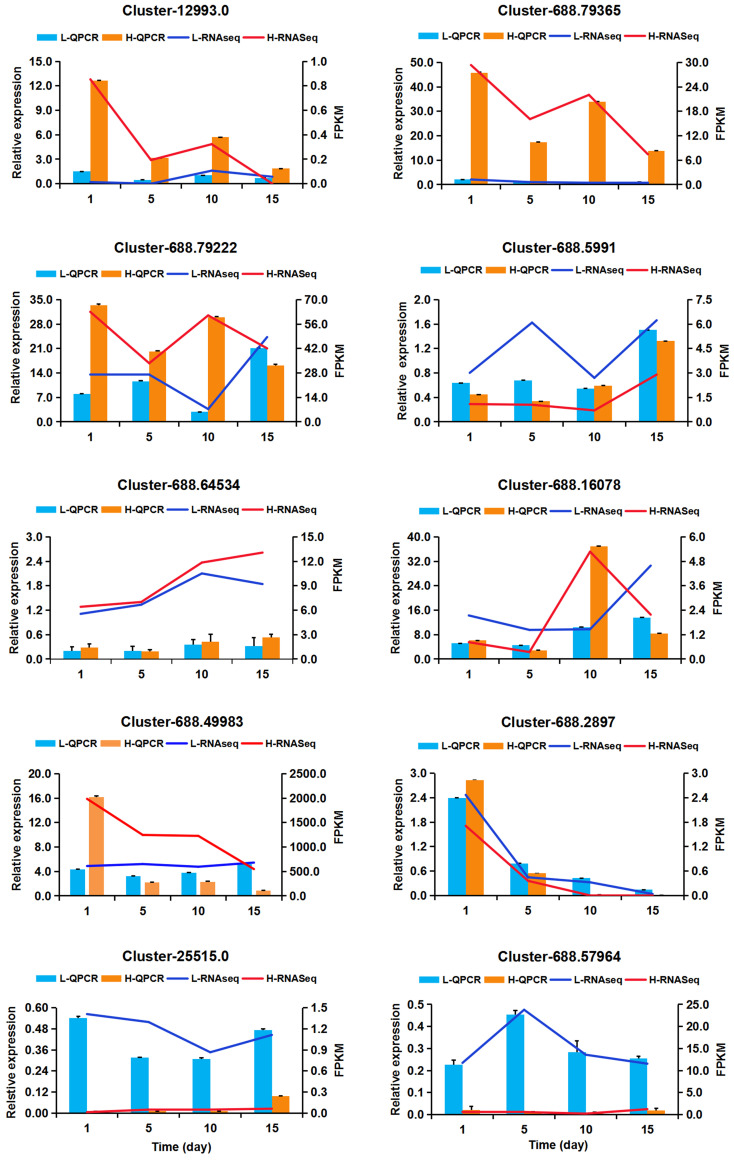
Quantitative real-time PCR validation of 10 DEGs. X-axis means NaCl treatment times. The left Y-axis represents the relative expression level of genes and displayed in bar charts, while the right Y-axis represents the PPKM value and showed by fold lines. Values are presented as the mean ± the standard error (SE) with three biological replicates.

In order to verify RNA-seq data, 10 genes involved in different important biological processes were selected for QPCR verification analysis. These 10 genes were involved in ABA signal transduction (Cluster-12993.0 encoding *PP2C* and Cluster-688.5991 encoding Abscisic acid receptor *PYL4*), peroxidase (Cluster-25515.0 encoding peroxidase 44-like), Glycerophospholipid metabolism (Cluster-688.49983 encoding phosphoethanolamine N-methyltransferase), transcription factors (Cluster-688.16078 encoding AP2/ERF-ERF and Cluster-688.2897 encoding C2H2), (Cluster-688.57964 encoding BURP domain-containing protein 5-like), and three genes, Cluster-688.64534, Cluster-688.79365, and Cluster-688.79222, highly expressed at all the treatments without any annotation information. The results of QPCR analysis are shown in **Figure 8**. Moreover, the expression correlation between RNA-seq data and QPCR was calculated, and the correlation coefficient was 0.8853 (**Supplementary Figure S8**), suggesting that the reliability of RNA-seq data was high and the DEGs obtained from RNA-seq data should be reliable.

## 4 Discussion

**Figure 9:**
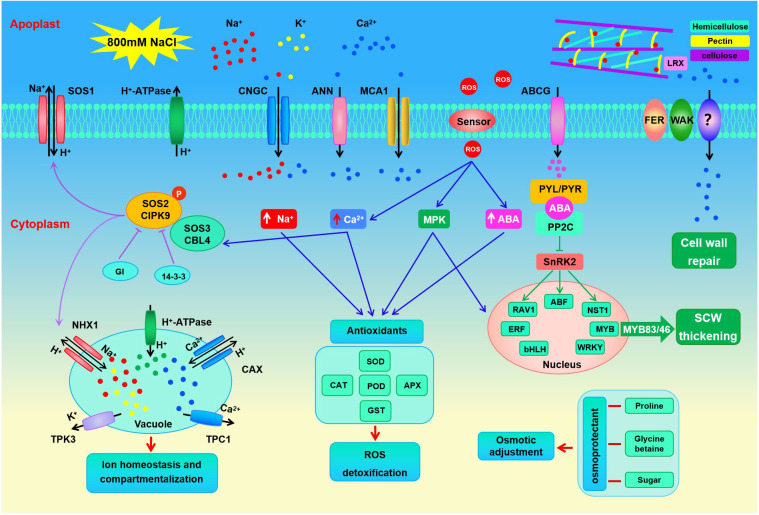
Putative mechanisms of halophyte *S. dendroides* plant adapt to high slat stress.

Soil salinization has become a global ecological problem, especially in arid and semi-arid areas, which affects the establishment, development, and growth of plants and eventually leads to crop yield reduction (Lesk et al., 2016; Kopecka et al., 2023). Halophytes grow well in saline soil with a concentration of about 200 mM NaCl or above and survive in extremely harsh environments (Flowers and Colmer, 2008), which provides valuable resources for us to study the complex physiological and molecular mechanisms of plants adapting to abiotic stress. In this study, the adaptive mechanisms of *S. dendroides* associated with high salt stress were analyzed at the physiological and molecular levels. The fresh weight, dry weight, and relative water content of seedlings showed the highest under 200 mM NaCl treatment (**Figures 1A-C**), indicating that a low concentration of NaCl could promote the growth of seedlings. Like many succulent euhalophyte species, for example, *Suaeda salsa* grows optimally at 200 mM NaCl, and the optimal growth is accompanied by an increase in succulence and other morphological changes (Song et al., 2009). Under 800 and 1000mM NaCl stress, the seedlings showed wilting symptoms, and some seedlings even died under 1000mM NaCl. However, the wilting symptoms of the seedlings began to recover after 5 days of the treatment, recovered after 10 days and gradually returned to normal growth. After 10 days of NaCl treatment, the content of Na and K in the shoots of *S. dendroides* showed that the change of Na/K ratio was moderate and kept a relatively stable level when the salt concentration increased to 600 mM (**Figures 1G-I**). These indicate that *S. dendroides* could maintain Na/K balance under high salt stress to avoid ion toxicity. Furthermore, high salt stress can induce the accumulation of proline and soluble sugar, providing osmoprotection and energy for *S. dendroides* plants to adapt to stress environments. The sequencing result showed that the DEG number was the highest after 10 days of high salt treatment (**Figure 2A**), indicating that this period was a crucial time for *S. dendroides* to adapt to the saline environment. According to the expression level of key genes at different treatment times of high salt treatment, our study indicates that cell wall remodeling, ion homeostasis and compartmentalization, osmotic adjustment, and plant hormone signal transduction regulate the adaptation mechanism of *S. dendroides* suffering from high salt stress (**Figure 9**).

### 4.1 Cell wall regulation of *S. dendroides* under high salinity

The plant cell wall is a highly dynamic and complex network, mainly composed of different polysaccharides, lignin, and various structural proteins, which is a barrier to protect plants from the environment (Vaahtera et al., 2019). The cell wall integrity (CWI) maintenance system plays a crucial role in stress sensing and response for plants (Vaahtera et al., 2019; Rui and Dinneny, 2020). At present, a range of plasma membrane-localized receptor-like kinases and cell wall glycoproteins involved in CWI perception and maintenance have been identified. *FER* belongs to *CrRLK1L* family and is regarded as a cell wall sensor, which is necessary to activate Ca^2+^ influx and maintain CWI under salt stress (Feng et al., 2018). Other protein kinase genes, such as *WAK* and *LRX*, are thought to bind to cell wall components and play an important role in regulating cell wall integrity (Decreux and Messiaen, 2005; Herger et al., 2019). In our study, 4 *FER*, 1 *LRX3*, 5 *WAK*, and 4 *WAKL* genes were differently expressed under 800mM NaCl stress (**Figure 5D**). Several proteins, enzymes, and ions are related to the cell wall modifications in response to abiotic stresses. Generally, salt stress may directly trigger the activation of pectin methyl esterase, which leads to the decrease of pectin methylation degree, thus affecting the characteristics of cell wall (Gigli-Bisceglia et al., 2022). Acetylation is another important pathway of pectin modification (de Souza et al., 2014). Homogalacturonan (HG) is the most abundant pectic polymer, and its remodeling is regulated by special enzymes, such as *PME*, *PAE*, *PG*, or *PLL* (Senechal et al., 2014). In *S. dendroides*, some cell wall modification related genes were identified, 4 *PME*, 5 *PL*, and 4 *PG1* genes were up-regulated under 800 mM NaCl stress (**Figure 5E**). The result showed that the enzymes involved in pectin modification might be important for *S. dendroides* to adapt to high salt stress. Additionally, cell wall-loosening protein EXPs and XTHs play important roles in coping with high salt stress. *AtXTH30* has a negative effect on salt tolerance (Yan et al., 2019). *AtXTH19* and *AtXTH23* were induced by brassinosteroids, and the double mutant (*atxth19atxth23*) was sensitive to salt stress (Xu et al., 2020). *EXPs* were induced by salt stress, and overexpression of *EXP* gene was helpful to promote plant salt tolerance (Chen et al., 2017; Jadamba et al., 2020). 20 *XTHs* and 3 *EXPAs* in *S. dendroides* were obtained and differently expressed under 800 mM NaCl stress (**Figure 5B**). Thus, cell wall remodeling is particularly important for *S. dendroides* to adapt to a high concentration of Na^+^ condition.

SCW is thickened cell walls, mainly consisting of cellulose, hemicellulose, lignin, a minor amount of structural proteins and enzymes, which play an essential role in maintaining plant morphology, providing rigid support and ensuring material transportation, and participating in plant biotic and abiotic stress response as a protective barrier (Cosgrove, 2005; Hamann, 2012). In this study, cellulose synthase genes *CESA4*, *CESA7*, and *CESA8*, related to the SCW synthesis, increased prominently after 10 and 15 days of high salt stress (**Figure 5A**). PPI analysis showed that the expression of *COBL4* was positively correlated with the expression of cellulose synthesis genes *CESA4*, *CESA7*, and *CESA8* related to SCW (**Figure 5F**). COBL4 encodes a COBRA-like protein that has typical structural characteristics of a glycosylphosphatidylinositol-anchored protein, like sorghum SbBC1, which is homologous to OsBC1 and AtCOBL4, involved in the biosynthesis of cellulose in SCW and affects the mechanical strength of sorghum plants (Li et al., 2019). The expression level of *CESA4*/*7*/*8* and *COBL4* increased significantly under 800 mM NaCl treatment, indicating that high salt stress can promote SCW cellulose synthesis. Xylan is the main component of hemicellulose polysaccharide. Genetic and biochemical analyses revealed that the synthesis of SCW xylan backbone was mediated by *IRX10*/*IRX10L* of the GT47 family, *IRX9*/*IRX9L* and *IRX14*/*IRX14L* of the GT43 family, and *GUXs* of the GT8 family (Zhong et al., 2019). As the result showed in **Figure 5A**, the DEGs involved in xylan backbone synthesis, such as *IRX9*, *IRX14*, *IRX15*, and *IRX15L*, were up-regulated and significantly increased after 10 and 15 days of 800 mM NaCl stress. Moreover, the expression level of the gene *IRX7* involved in xylan reduction terminal synthesis and the genes *GUX1* and *GUX2* involved in side chain synthesis increased significantly after 10 and 15 days of high salt stress. In addition, 5 *TBL* encoding DEGs were significantly up-regulated after 15 days of 800mM NaCl stress. *TBL32* and *TBL33* are putative acetyltransferases, which participate in acetyl substitutions of 2-O-GlcA-substituted xylosyl residues, playing important roles in xylan acetylation and normal deposition of the SCW (Yuan et al., 2016). Furthermore, PPI network analysis of DEGs at different treatment times under high salt stress showed that the hub gene *IRX9* (Cluster-688.80914) was positively correlated with *GUX1*, *IRX7*, *CSEA4*, *CESA7*, *CESA8* and *SUS3*. These indicated that high salt stress could promote the synthesis of xylan in SCW of *S. dendroides*.

However, the coordinated expression of genes involved in cellulose, xylan, and lignin biosynthesis in SCW is mediated by the NAC-MYB transcriptional network (Nakano et al., 2015). A set of closely related NAC domain TFs acts as the primary switch of plant SCW biosynthesis, activating downstream TFs to regulate the entire network of SCW biosynthesis (Mitsuda et al., 2005; Mitsuda et al., 2007). Most of the R2R3-MYB subfamily TFs function as the second layer-master switches of SCW biosynthesis, such as *MYB26*/*103* and *MYB46*/*83* (Xiao et al., 2021). Moreover, the genes involved in ABA synthesis and signal transduction are related to SCW thickening and lignification in *Arabidopsis* (Endler and Persson, 2011; Wang L. et al., 2020). *AtNST1*, a NAC domain family TF that can activate the downstream genes related to SCW biosynthesis. Studies have shown that *AtNST1* is a key phosphorylation substrate of *SnRK2*, which acts as the key positive regulator of ABA signal transduction, which regulates SCW formation and lignin deposition in the stem fiber region of *Arabidopsis thaliana* (Liu et al., 2021). In this study, the expression level of gene *NST1* was significantly increased after 15 days of 800 mM NaCl stress (**Figure 7D**, **Supplementary Table S7**). *MYB4*, *MYB86*, and *MYB26*/*103* were significantly up-regulated after 15 days of high salt stress, and *MYB46*/*83* was notably up-regulated after 10 days (**Figure 7D**). Therefore, the genes involved in SCW biosynthesis and thickening may be extremely important for *S. dendroides* adapting to high salt environment. In brief, multiple genes related to cell wall biosynthesis, modification, sensing, and CWI maintenance of *S. dendroides* were significantly expressed under high salt stress. The results indicated that cell wall remodeling and SCW thickening were the key drivers for *S. dendroides* plants adapting to high salt stress.

### 4.2 An efficient ion transport system is essential for *S. dendroides* response to high salt stress

Succulence is another mechanism for euhalophyte plants to adapt to high salt environments. A large amount of inorganic ions and organic osmotic adjustment substances are accumulated in succulent leaves and stems to maintain osmotic balance (Flowers and Colmer, 2015; Rahman et al., 2021). In this study, a series of genes related to inorganic ion transport, including *SOS1*, *HKT1*, *NHX1*, *TPK3*, *PMA*, and *AVP*, were prominently expressed under 800 mM NaCl treatment (**Figure 6A**, **Supplementary Table S7**). The plasma membrane Na^+^/H^+^ antiporter *AtSOS1* is located at the root tips, excluding Na^+^ from the root, thus reducing Na^+^absorption (Shi et al., 2000). Na^+^ influx into cells is mediated mainly by non-selective cation channels and the sodium transporter *HKT1*. It has been proved that *AtHKT1* can unload Na^+^ from xylem vessels to parenchyma cells and/or control the retrieval of Na^+^ from xylem to reduce the amount of Na^+^ transported to shoots (Sunarpi et al., 2005). In addition, *SOS1* and *HKT1* mediate opposite Na^+^ fluxes, and coordinate the regulation of Na^+^ transport and ion homeostasis under salt stress (Wang W.-Y. et al., 2020). Tonoplast located Na^+^(K^+^)/H^+^ antiporter *NHX1* has been proven to separate excessive cytoplasmic Na^+^(K^+^) into vacuoles, which was motivated by the proton motive force produced by vacuolar H^+^- ATPase (*VHA*) and H^+^-PPase (Yang et al., 2010). The *KT*/*HAK*/*KUP* family genes act as K^+^/H^+^ symporter, and most of them are important for K^+^ absorption in plant roots (Wang Y. et al., 2021). Tonoplast localized *AtTPK1* functions in K^+^ transport across the tonoplast and plays important roles in many physiological processes (Gobert et al., 2007). As a representative euhalophyte, *S. dendroides* could accumulate a large amount of Na^+^ in succulent leaves, and maintain the stability of K^+^ by promoting the ability of K^+^ transportation under high salt treatment, indicating that *S. dendroides* has the ability to modulate Na^+^/K^+^ balance. Moreover, it has been found that the concentration of cytosolic Ca^2+^ ([Ca^2+^]cyt) increases with numerous environmental conditions. Constant [Ca^2+^]cyt is maintained by Ca^2+^ influx through Ca^2+^-permeable ion channels or transporters located in the plasma membrane or tonoplast, and other membranes. *MCA1* and *MCA2* are responsible for Ca^2+^ influx in *Arabidopsis* cells. Over-expression of *MCA1* driven by a strong promoter can promote the absorption of Ca^2+^ in roots and increase cytoplasmic Ca^2+^ concentration upon hypoosmotic shock (Yamanaka et al., 2010). Two-pore calcium channel protein TPC1 located in the tonoplast acts as a non-selective cation channel, which is co-regulated by voltage and Ca^2+^ to produce a slow vacuolar current (Ye et al., 2021). besides, *CNGC*, located mostly in the plasma membrane, enables the absorption of Na^+^, K^+^, and Ca^2+^. *AtCNGC10* is involved in K^+^ and Na^+^ absorption and long-distance transportation, and *AtCNGC17* mediated the influx of osmotically active K^+^ and the second messenger Ca^2+^, or both (Jarratt-Barnham et al., 2021). In *S. dendroides*, 3 *CNGC* genes were significantly expressed, *CNGC4* was significantly increased, while 2 *CNGC17* genes decreased at all the time points of 800 mM NaCl treatment (**Figure 7A**, **Supplementary Table S7**). Furthermore, cytosolic Ca^2+^ binds to calcium sensor protein SOS3/CBL4, and subsequently interacts with protein kinase *SOS2*/*CIPK24* to form a complex activator that activates SOS1, a Na^+^/H^+^ antiporter, resulting in Na^+^ efflux from the cytosol. The salt overly sensitive (SOS) pathway comprises *CBL4*/*SOS3*, *CIPK24*/*SOS2*, and *SOS1*, which has been considered the key mechanism of Na^+^ homeostasis and salt tolerance in *Arabidopsis* (Zhu, 2000). Apart from interacting with SOS1 at the plasma membrane, *CIPK24* is also reported to regulate the activity of several tonoplast located transporters by interacting with them, such as Ca^2+^/H^+^ antiporter (Cheng et al., 2004), vacuolar V-ATPase (Batelli et al., 2007), and Na^+^/H^+^ exchanger (Qiu et al., 2004; Huertas et al., 2012). In this study, *CIPK9*, the closest *SOS2* homolog in *S. dendroides*, functions in a similar manner to *SOS2* in the context of the *SOS3*-*CIPK9*-*SOS1* complex to mediate salt tolerance in halophytes *S. dendroides* (**Figure 9**). These indicate that *S. dendroides* has an efficient Na^+^ transport and compartmentalization mechanism to maintain ion homeostasis under high salt environment.

In addition to Na^+^/K^+^ homeostasis, higher plants need to adjust the balance of various nutrients in saline environments. Several *Arabidopsis* NPF genes were discovered to play a crucial role in nitrate uptake and transport (Corratge-Faillie and Lacombe, 2017), such as *NRT1.1*/*AtNPF6.3/CHL1* involved in nitrate uptake. *NPF7.3*/*NRT1.5* plays important roles in K^+^ translocation from root to shoot, and it also participates in coordination of K^+^/NO_3_^-^ distribution in plants. *NPF2.5* was expressed in the plasma membrane of root cortical cells, functioned in Cl^−^ efflux from the root, and was up-regulated by NaCl. In the present study, the DEGs involved in the absorption and transportation of some important nutrients were significantly induced by high salt stress. Notably, 15 NPF encoding genes were identified and differently expressed under high salt treatment, in which 7 *NPFs*, including 4 *NPF 3.1* and *NPF 1.1*/*1.2*/*6.4*, were significantly increased after 1 day of high salt stress, while 8 NPFs were down-regulated (**Figure 6A**, **Supplementary Table S7**). In addition, *NPF4.6*/*AIT1*/*NRT1.2* functions as an ABA importer at the site of ABA biosynthesis, plays an important role in regulating the stomatal aperture in inflorescence stems (Kanno et al., 2012). Moreover, it has been found that several members of the ABCG subfamily functioned in ABA transport (Borghi et al., 2015), such as *ABCG25*, which functions as an ABA exporter from the vascular tissue, and *ABCG40* is an ABA uptake transporter in guard cells. A total of 18 ABC family genes were differentially expressed under 800 mM NaCl treatment, including 2 *ABCBs*, 11 *ABCCs*, and 5 *ABCGs*, among which the genes of the ABCC subfamily and the ABCG subfamily were more active under high salt stress (**Figure 6A**, **Supplementary Table S7**). Therefore, *S. dendroides* could promote the absorption and transport of various nutrient elements by increasing the transcription lever of transporters/channels.

### 4.3 Organic osmolyte accumulation is indispensable for *S. dendroides* adaptation to high salinity

PPI analysis showed that *P5CS* interacted with the DEGs encoding *ALDH* and *BADH*, genes involved in Ca^2+^ signaling and antioxidants (**Figure 6C**). The ALDH superfamily comprises a variety of enzymes involved in endogenous and exogenous aldehyde metabolism (Tola et al., 2021). *P5CS*, a rate-limiting enzyme in proline biosynthesis, is a member of ALDH18 family, and its expression is significantly up-regulated in dehydration reactions (Yoshiba et al., 1997). Studies have shown that the accumulation of proline under salt stress is related to the up regulation of *P5CS* (Mansour and Ali, 2017). Proline plays an essential role in protecting plant growth from unfavorable environmental conditions by osmotic adjustment, working as a molecular chaperone, and protecting the integrity of proteins and enzymes (Ozturk et al., 2021). Nevertheless, phytohormone ABA is a crucial component in integrating multiple signals, controlling downstream stress responses, and influencing proline synthesis and accumulation by regulating the expression of *P5CS* and *P5CR* in plants (Verslues and Bray, 2006). The interaction of ABA and the Ca^2+^ signaling pathway can also regulate proline accumulation in plants under stress conditions. In this study, several DEGs encoding *TPC1A*, *MCA1*, *CBL4*, and *CML22* involved in Ca^2+^ signaling pathway were directly or indirectly related to *P5CS* (**Figure 6C**). In addition, proline can also stabilize the antioxidant system and protect the integrity of cell membranes through osmotic adjustment, thereby reducing the influence of ROS and overcoming or repairing stress injury (Banu et al., 2009). In *S. dendroides*, the major antioxidant enzymes, including *SOD*, *CAT*, *APX*, *CPR*, and *Fer*, obviously expressed under high salt stress, may interact with the genes involved in osmoregulation (**Figure 6C**). *BADH*, also known as *ALDH10* family genes, has been widely studied for its role in stress responses and the production of the osmoprotectant GB (Tola et al., 2021). In plants, *PEAMT* is the rate-limiting enzyme for synthesizing choline, the precursor of betaine (Nuccio et al., 2000), while CMO and BADH are the key enzymes involved in betaine biosynthesis. It was found that the tolerance of gene *CMO* and *BADH* over-expressed plants was enhanced (Shirasawa et al., 2006; Wu et al., 2008). In our study, several key genes involved in GB synthesis were significantly up-regulated under 800 mM NaCl stress, like *CMO*, *BADH*, and *PEAMT* encoding genes were continuously expressed under 800 mM NaCl treatment for 1, 5, 10, and 15 days (**Figure 6B**). The result above suggests that *S. dendroides* could synthesize and accumulate a large number of osmoprotectants to protect the plants from high salt stress by regulating the expression patterns of key genes involved in the biosynthesis of compatible solutes.

## 5 Conclusion

This study identified potential genes showing a possible adaptation mechanism of halophytic *S. dendroides* encountering a high salt condition, mainly involved in cell wall metabolism, inorganic ion transport, and organic osmolyte synthesis (**Figure 9**). A large number of DEGs encoding cell wall sensing, modification, and secondary wall thickening were prominently increased under 800 mM NaCl treatment, which might be important for cell wall repair and integrity maintenance under high salt concentrations. Moreover, the DEGs encoding Na^+^, K^+^, Ca^2+^, and Cl^-^/NO_3_^-^ transporters/channels were significantly increased under 800 mM NaCl treatment, which might be beneficial to promote the absorption and transport of nutrient elements under high salt stress. Notably, some genes involved in Na^+^/K^+^ transport (such as *SOS1*, *HKT1*, *NHX*, and *TPK3*) might play important roles in Na^+^ transporting and sequestering, maintaining ion homeostasis under high saline conditions. Moreover, several genes involved in the synthesis of organic regulators were significantly up-regulated under 800 mM NaCl treatment, suggesting that *S. dendroides* possesses an effective mechanism to accumulate more osmoprotectants to enhance salt tolerance. Our results contribute a new perspective for understanding the molecular mechanisms of halophytes adapting to high salinity and provide a basis for genetic improvement of salt tolerance in important plants by using excellent genes from halophytic *S. dendroides*.

## Data availability statement

The datasets presented in this study can be found in online repositories. The names of the repository/repositories and accession number(s) can be found below: SRA, and the accession numbers PRJNA1018260.

## Author contributions

PM: Conceptualization, Methodology, Supervision, Writing – original draft, Writing – review & editing, Formal Analysis, Investigation. JL: Methodology, Writing – review & editing. GS: Methodology, Conceptualization, Project administration, Writing – review & editing, Funding acquisition, Investigation, Resources, Supervision. JZ: Conceptualization, Methodology, Project administration, Writing – review & editing.
