# Supplementary material for: Comparative transcriptome analysis reveals the adaptive mechanisms of halophyte Suaeda dendroides encountering high saline environment
Source: Front Plant Sci. 2024 Feb 14;15:1283912. doi: 10.3389/fpls.2024.1283912 (PMC10899697; doi:10.3389/fpls.2024.1283912)
Supplement: Supplementary file 2 [file DataSheet_1.pdf]

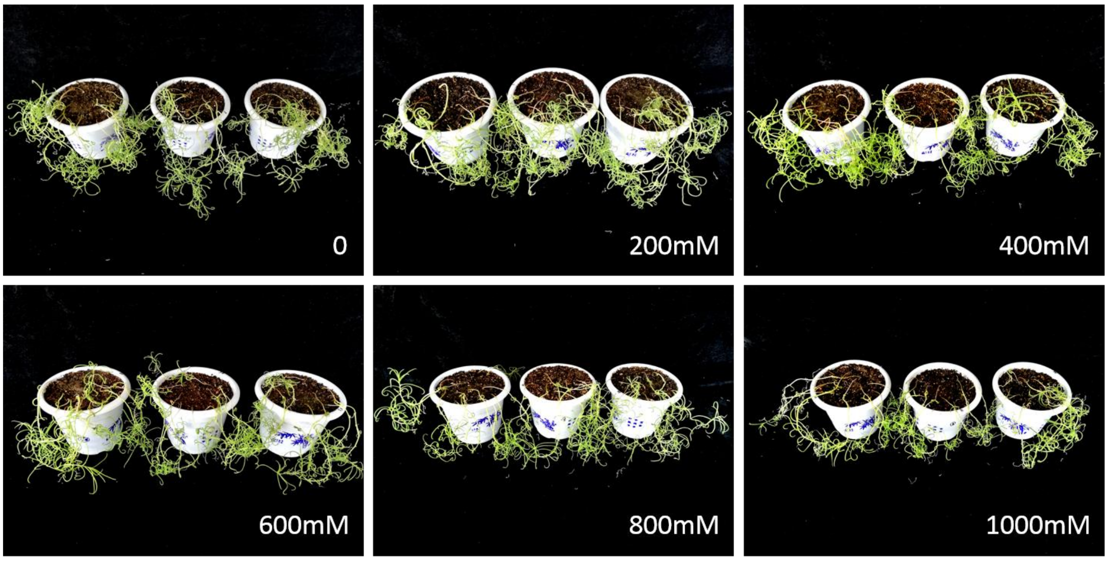

Figure S1 The growth of *S. dendroides* under different salt concentrations

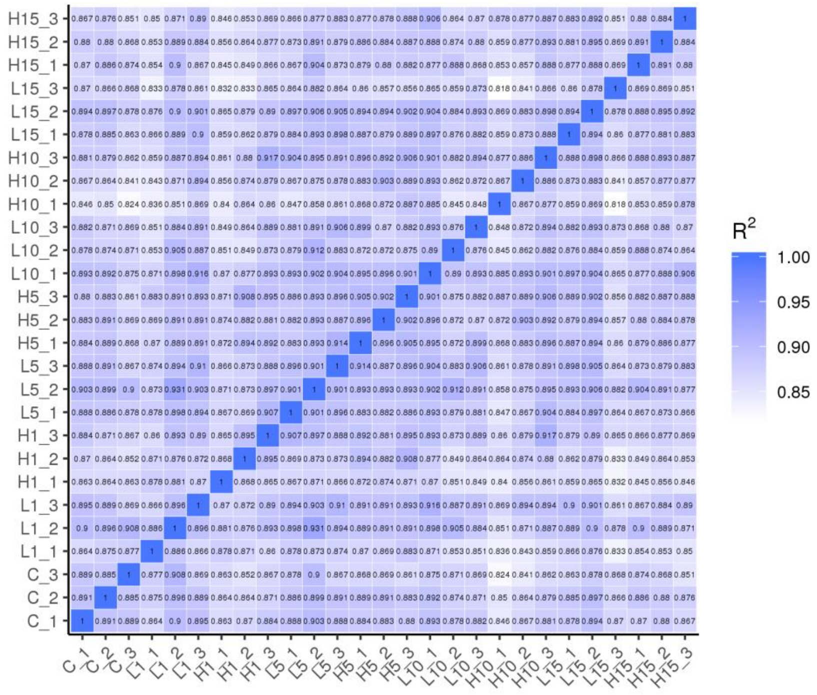

Figure S2 Pearson coefficients between samples

The x-axis represents the log10 (FPKM+1) of sample 1, and the y-axis represents log10 (FPKM+1) of sample 2, R2: the square of Pearson correlation coefficient. Different columns represent different samples, and different rows represent different genes. The color represents the base 2 logarithm of the FPKM expression level of the gene in the sample. Color from red to green means the correlation gradually increased. Clustering together with similar correlations.

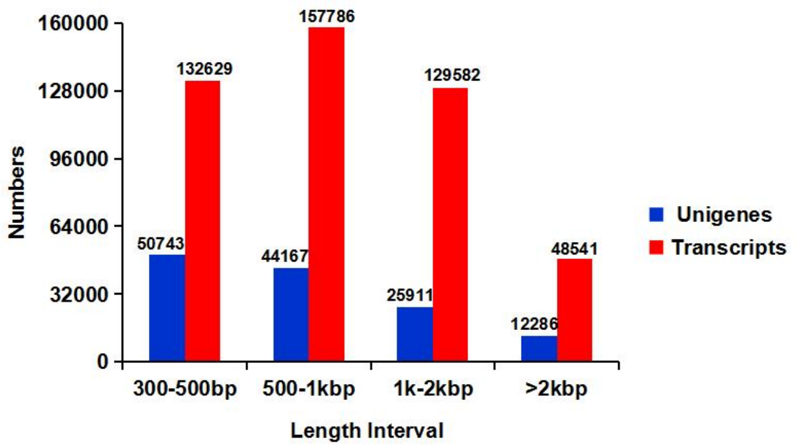

Figure S3 A length distribution of the transcripts/unigenes generated via de novo assembly.

The x-axis represents the All-transcripts/Unigenes lengths, and the y-axis represents the number of All-transcripts/Unigenes in a certain length range.

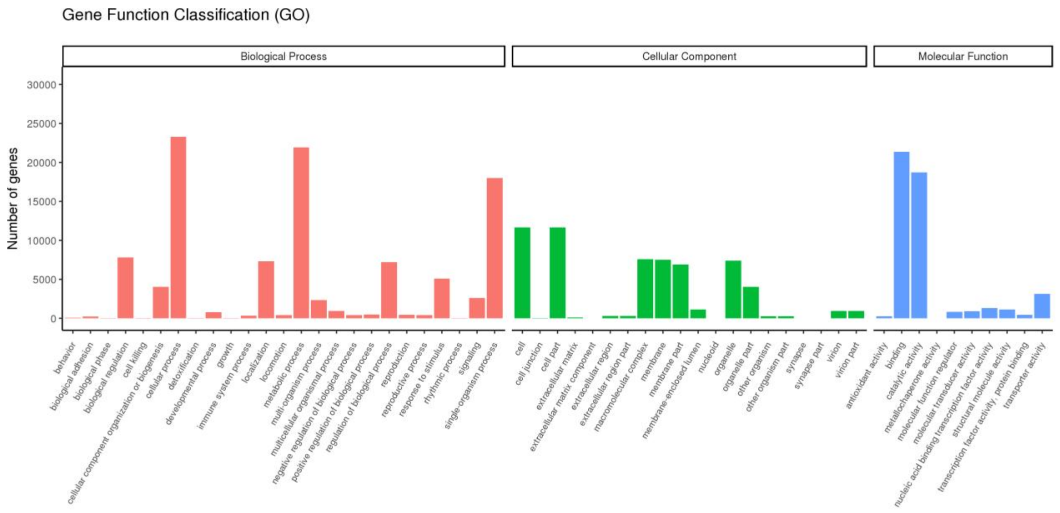

Figure S4 The gene ontology (GO) annotations of all annotated unigenes

These results were summarized under the three main GO categories: biological process, cellular component and molecular function. The left Y-axis indicates the percentage of a specific category of genes in the corresponding GO category.

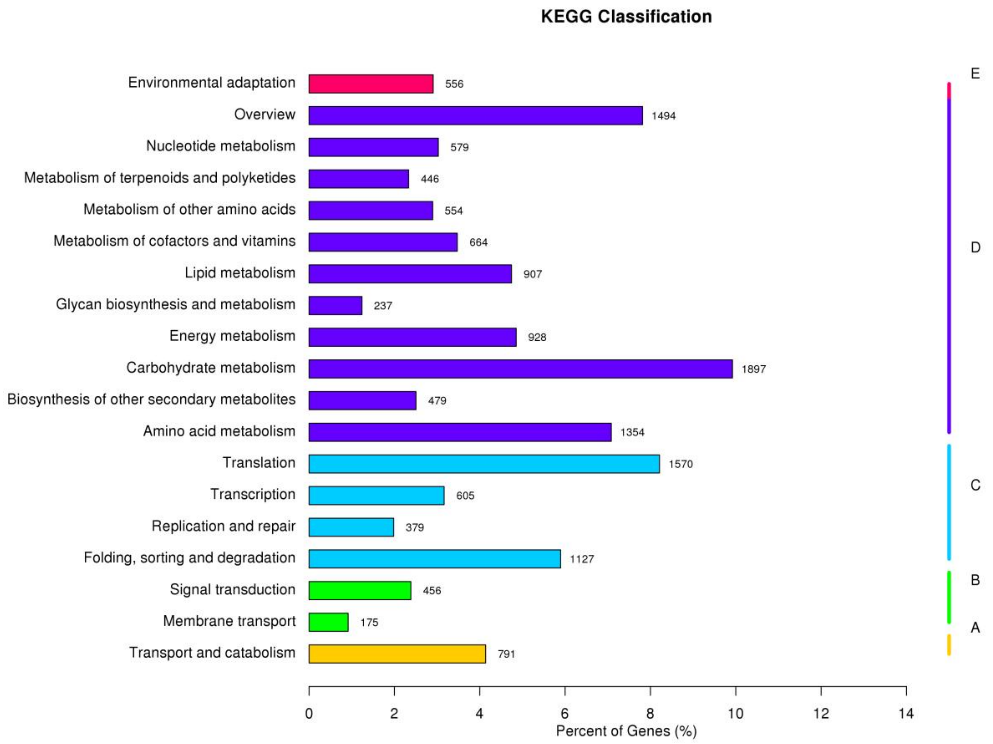

Figure S5 KEGG function Classification of unigenes

The ordinate in the left represents the percentage of the number of genes, the right ordinate represents the number of unigenes. The abscissa is the classification of KEGG.

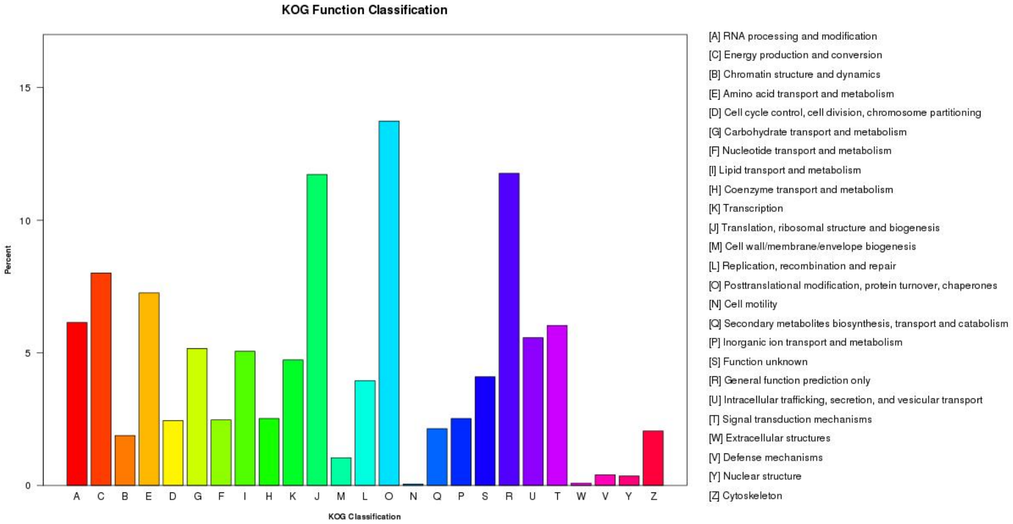

Figure S6 Clusters of KOG annotations of unigenes

The Y-axis indicates the number of unigenes in a specific functional cluster.

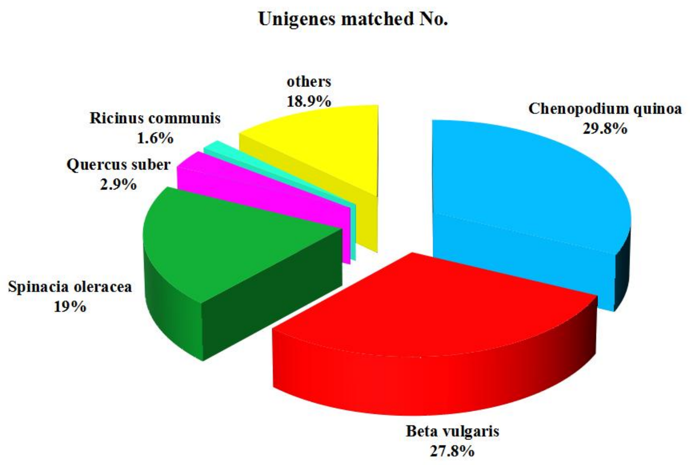

Figure S7 Species Classification of unigenes

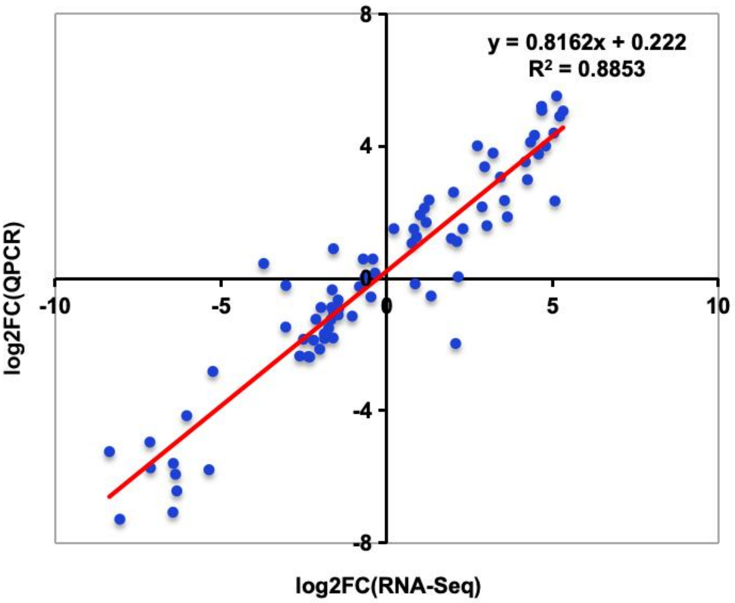

Figure S8 Correlation between RNA-Seq expression profile and QPCR results
